# Zinc Oxide Nanoparticles as Diagnostic Tool for Cancer Cells

**DOI:** 10.1155/2022/2807644

**Published:** 2022-11-02

**Authors:** Sumayah Ibraheem, Afraa Ali Kadhim, Kadhim Ali Kadhim, Ihssan A. Kadhim, Majid Jabir

**Affiliations:** ^1^Al_kindy College of Medicine, University of Baghdad, Baghdad, Iraq; ^2^Department of Biology, College of Science, Mustansiriyah University, Baghdad, Iraq; ^3^Department of Pharmacy, Al-Yarmok University College, Baghdad, Iraq; ^4^Ministry of Science and Technology, Baghdad, Iraq; ^5^Applied Science Department, University of Technology, Baghdad, Iraq

## Abstract

ZnO nanoparticles have various characteristics that make them attractive to be used in many medical applications like a cancer diagnosis. It can be used as a nanoprobe for targeting different types of cancer cells in vitro as a cancer cell recognition system. The present study aims to investigate the permeability of ZnO NPs through both normal and cancerous cell lines in humans. In vitro experiments for ZnO NPs inside the environment of living cells have been described, which would contribute to the visualization of nanoparticles as cancer diagnostic and scanning techniques. MCF7, AMJ13, and RD cancer cells, and also the normal breast cell line HBL, were used in in vitro imaging experiments. The findings revealed that ZnO NPs specifically incorporated within tumor cells while accumulating less inside normal cells. Our findings show that ZnO NPs may be identified inside cancer cells after 1 h of exposure and can endure up to 3 h, providing them appropriate for tumor cell imaging. The findings showed that ZnO NPs might be employed as an alternate fluorophore for diagnostic imaging in the early identification of solid cancers. Therefore, here we studied in vitro applications of ZnO NPs and their beneficial use as a diagnostic tool for cancer cell lines rather than normal cells. Taken together, ZnO NPs can be used as good targeting NPs for the development of imaging agents for early diagnosis of cancers.

## 1. Introduction

Nanoparticles are often used as a vector for carrying bioactivities within tissues [1]. Among the several forms of nanoparticles, zinc oxide nanoparticles (ZnO NPs) are commonly employed. ZnO NPs are used in biosensors and photodetectors, and UV absorbers in beauty products, solar cells, thermoelectric, and antivirus medicines against many viral infections, and also in dyes and cosmetics [2]. ZnO is among the greatest semiconductor compounds, providing an excellent substitute for conventional Cd-associated substances used in biological applications and ophthalmology [3]. The in vitro study by Choudhary et al. [4] employed carbon nanoparticles in the internal monitoring of ZnO nanoparticles through MCF-7 breast carcinoma using the confocal imaging method. The unique physicochemical characteristics of nanoparticles have the potential to change imaging technologies [5]. Because of the asymmetric configuration of ZnO NPs, they may be employed as unsonorous nonlinear absorption probes using secondary harmonics generation (SHG) approaches [6]. Amongst the greatest attractive features of ZnO NPs considering utilization, bio-imaging applications are both biodegradable characteristics and weak toxicity [7]. Using their inherent fluorescence, ZnO NPs were visualized in vitro and in vivo in skin penetration [8]. This inherent fluorescence property benefits cytology and histology microscopy examinations by eliminating the requirement for external dyes. Additionally, visual scanning with ZnO nanomaterial allows easier experimental animal surveillance [7]. The present study aims to investigate the permeability of ZnO NPs through both normal and cancerous cell lines in humans. The results of the current study demonstrated that directed ZnO NPs permeate and aggregate in tumor cells having great specificity, and the fluorescence intensity was stronger than that of normal cells. In vitro experiments for ZnO NPs inside the environment of living cells have been described, which would contribute to the visualization of nanoparticles as cancer diagnostic and scanning techniques.

## 2. Materials and Methods

### 2.1. Materials and Reagents

The cell lines MCF-7 and AMJ13, the human rhabdomyosarcoma (RD), and the normal beast tissue cell line (HBL) were gifted from ICCR (Baghdad/Iraq). Dulbecco's Modified Eagle's Medium (DMEM) (Usbiological, USA), fetal bovine serum (FBS), penicillin, streptomycin, and trypsin-EDTA were purchased from (Capricorn- Scientific, Germany). Zinc acetate dehydrates and NaOH were purchased from Sigma-USA.

### 2.2. Preparation of ZnO NPs

The preparation of ZnO NPs was done by dissolving 4.16 g of zinc acetate dehydrates in 100 ml of deionized water and shaking it for 30 min. Accordingly, 3.5 g of NaOH was dissolved in 100 ml of deionized water and shaken for 30 min. The mixture was then introduced by dropping to the previously prepared zinc acetate solution to produce a pH close to 12. After shaking the solution for 60 min, a precipitate was produced. The precipitate was rinsed three times before being filtered then drying the material for one day was carried out using an oven at 70°C. The precipitate was ground and stored for characterization.

### 2.3. Characterization of ZnO NPs

Manufacturing ZnO NPs were characterized using TEM (Philips EM) evaluations to evaluate particle size and shape, Dynamic light scattering (DLS) to investigate particle size distribution, and finally, AFM analysis for morphonology surface techniques. These techniques were primed by dropping the solution on silicon and leaving it to dry at 25°C before imagining it with Atomic force microscopy.

### 2.4. Culturing of Mammalian Cells

The breast cancer cell lines included MCF-7 and AMJ13, the human Rhabdomyosarcoma (RD), and the normal beast tissue cell line (HBL) was employed in this study. These cell lines were grown in Dulbecco's Modified Eagle's Medium (DMEM) (Usbiological, USA), which contained 10% fetal bovine serum (FBS), 100 units/mL penicillin, and 100 g/mL streptomycin (Capricorn- Scientific, Germany). The cell lines were initially maintained as adhesive monolayers growth inside a humidity incubator with 5% CO2 and a temperature of 37°C. After short trypsinization with trypsin-EDTA (Capricorn-Scientific, Germany), cells were collected. These cells are tested and verified on a regular basis [9].

### 2.5. Short Time Highest Acceptable Dose

Using 96-well microtiter plates, a quantitative crystal violet cell viability investigation was carried out to determine the highest acceptable safety doses for the (ZnO NPs) (Bio-World, USA). After 24 h of the incubation period, the monolayers were carried out and planted at a density of 10000 cells/well. In a serum-free medium, cells were treated to (ZnO NPs) at two-fold dilutions (50000 ug to 50 ug). After 4 h of treatment, cell viability was evaluated by applying 50 l of Crystal violet (Sigma Aldrich, USA) after eliminating the tissue culture media and incubating for 2 h at 37°C. It was rinsed with PBS once the stain was removed. The absorbency was measured using an ELISA reader (Biochrom, UK) at 492 nm (test wavelength) in triplets. The results were reported as a percentage of multiplication in comparison to vehicle-treated cells [10].

### 2.6. Structural Investigation and Quantification Image Assessment

To illustrate the targeting properties of ZnO NPs within tumors, the highest allowable dosages (150, 100, and 50 ug/ml) were utilized to treat the tumor cells AMJ13, MCF-7, and RD, as well as normal cells HBL. The treated cells were cultured over 1 h before being rinsed three times using PBS and examined under an inverted microscope (Micros, Austria), and shot with a digital camera (Micros, Austria).

### 2.7. Images Quantitative Analysis

Applying a light microscope (Leica-microsystems, Germany) and a camera, the cytogenetic sections were shot at _200 power in multiple randomized sections (Leica-microsystems, Germany). Image J (https://rsb.info.nih.gov/ij/) was used to analyze the images. The program detected and measured cells that were positive toward ZnO NPs. In data methods, each image was quantitatively measured in minimum 3 times, and statistically, analysis was performed utilizing GraphPad software and the one-way ANOVA test [11].

### 2.8. ZnO NPs Fluorescence Determination

The tumor cells which included AMJ13, MCF-7, and RD were employed for comparison to noncancerous breast cells HBL to determine the cancer cell focusing properties of ZnO NPs using their intrinsic fluorescence. The earlier cytotoxicity test investigation determined the appropriate dose. During a period of 4 h, most treatment cells accepted dosages of 50, 100, and 150 ug/ml. The cells were plated at 10000 cells/well and maintained for 24 h in 10% FCS growth media. To decide the best dosage of ZnO NPs, three maximum concentrations of 50, 100, and 150 ug/ml were given to monolayer cells both with and without DMSO. The fluorescence signals of the cells were obtained at different period intervals following treatment (i.e., 60 min, 90 min, 120 min, 150 min, 180 min, 210 min.) utilizing a FLUO star Omega Fluorescence microplate reader (BMG Labtech, Germany), to select the proper incubating duration (excitation, 485 nm; emission, 520 nm). This was accomplished upon the designated time points by rinsing the wells multiple times with PBS to eliminate noninternalized nanoparticles prior to the fluorescence read.

### 2.9. Statistical Analysis

The results of this research are reported as means standard error of the mean, and statistical analyses were carried out using the GraphPad Prism 5 software program (GraphPad Software, Inc. San Diego, California) [12, 13].

## 3. Results and Discussion

### 3.1. Characterization of Manufacturing ZnO NPs

**Figure 1:**
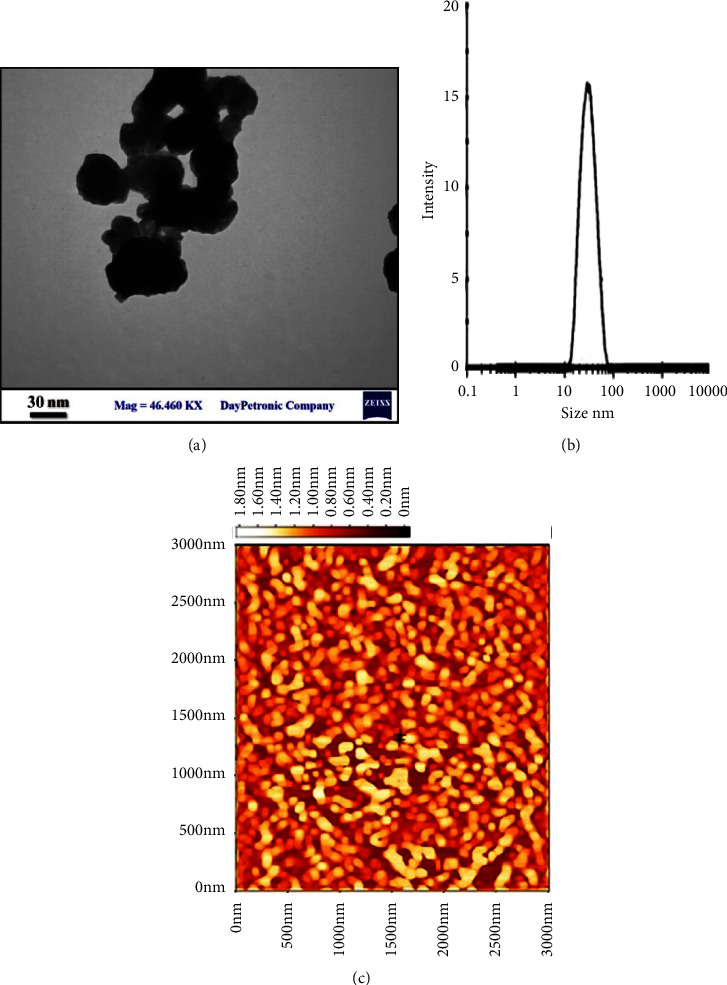
Characterization of ZnO NPs. (a) TEM. (b) DLS. (c) AFM histogram image showing uniformly distributed nanoparticles with spherical shape.

The TEM technique was used to measure compound size and shape.  Figure 1(a) shows the TEM characterizations for our produced ZnO NPs. The TEM revealed a rather circular shape of nanoparticles with diameters ranging from 10 to 20 nm.  Figure 1(a) exhibits TEM for ZnO NPs generated under optimal conditions. The creation of ZnO NPs is confirmed by TEM images, with a mean range of around 20 nm. TEM examination was performed to validate the real particle size, growth patterns, and crystallite dissemination. One of the most essential criteria is the main dimension of zinc oxide nanoparticles. Photon association spectroscopic or semi light dispersion are some other names for it. It is a quantum mechanics approach for determining the size spread characteristic for tiny particles in suspension or polymers in solutions. It may similarly be applied to understanding the characteristics of complicated fluids like saturated polymers. It is applied to determine the size of particles such as polymers, polysaccharides, micelles, or nanoparticles. The average functional size of the particles may be calculated if the environment is monodisperse. This determination is affected by the particle core size, structural size, particle concentrations, or ion class in the liquid. The diffusion coefficient of the particles may be measured because it simply monitors changes in light scattering challenges that affect by the diffusion of the particles. It is used to investigate particle stabilization. It also shows if the particles agglomerate across duration by observing if the particle's hydrodynamic radius increases. If nanoparticles agglomerate, a greater collection of particles with greater diameter will form. DLS observations revealed that particles were polydispersed, which implies that particles of various sizes were generated, i.e., the majority of particle size was around 20–30 nm (Figure 1(b)).  Figure 1(c) shows the topographic picture of well ZnO NPs generated under optimal conditions using AFM. It definitely shows the production of circular ZnO NPs. Nanometer-scale ZnO crystals were seen in the AFM picture. The photos demonstrate that the zinc oxide nanoparticles were tiny in size.

### 3.2. Structural Investigation and Quantification Image Assessment

**Figure 2:**
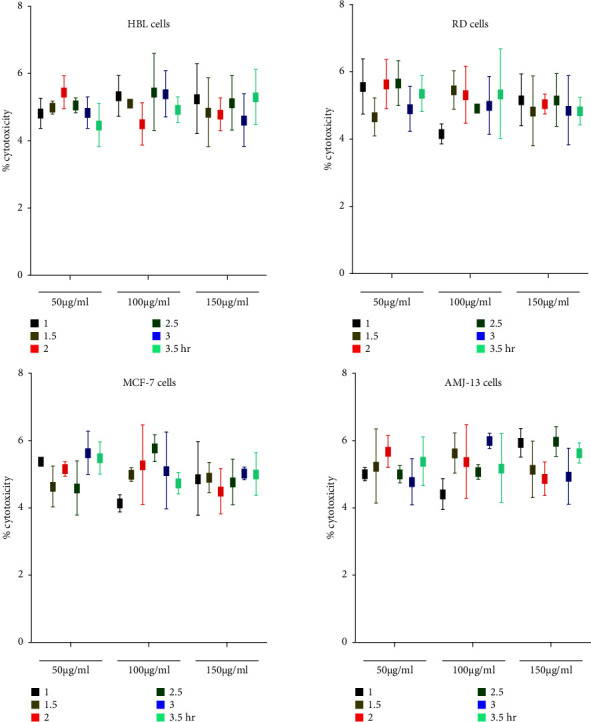
ZnO NPs cytotoxic activity against cancer and normal cell line. Data in the MTT test are represented by mean ± S.E.

**Figure 3:**
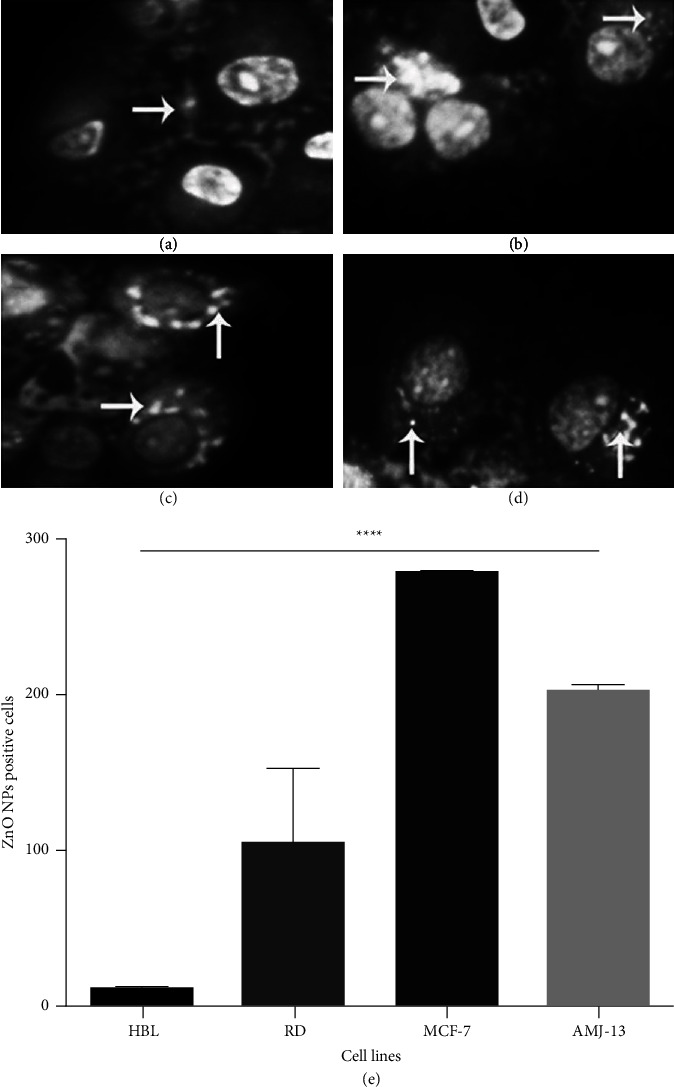
Morphological investigation of treated tumor and normal cells. (a) The normal cells of the breast did not accumulate ZnO NPs in treated cells. ZnO NPs internalized and aggregated within most tumor cells as illustrated (white arrow) from 1 h to 3.30 h in cells (b, c, d) RD, MCF7, and AMJ13. (e) The quantified data reveal a substantial difference in the concentration of ZnO NPs within tumor cells versus normal cells.

To analyze the impact of ZnO NPs on the viability of normal and tumor cells under various levels and times stated in Figure 2 MTT was used in this analysis. Cells treated to various doses of ZnO NPs exhibited no cytotoxic effects, thus findings demonstrate that the highest acceptable dosage surpassed 150 ug/ml. Morphometric observations revealed that ZnO NPs absorbed and accumulated within the tumor cells between 1 and 3.30 h, although mostly nontumor cells did not, as illustrated in Figure 3. The quantification study reveals that ZnO NPs accumulate significantly within tumor cells, but normal cells exhibit little or no accumulation.

### 3.3. ZnO NPs Fluorescence Determination Assay

**Figure 4:**
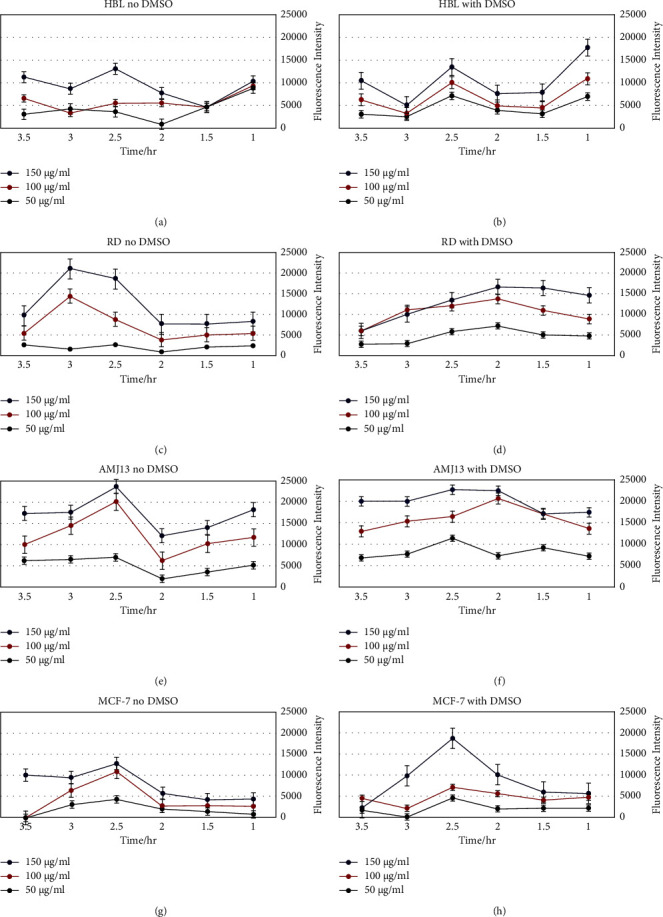
The impact of ZnO NPs levels on imaging (150 and 100 ug/ml is higher than 50 ug/ml). As contrasted to tumor cells, normal cells (HBL) exhibit lower fluorescence intensity to be identified throughout all time points.

**Figure 5:**
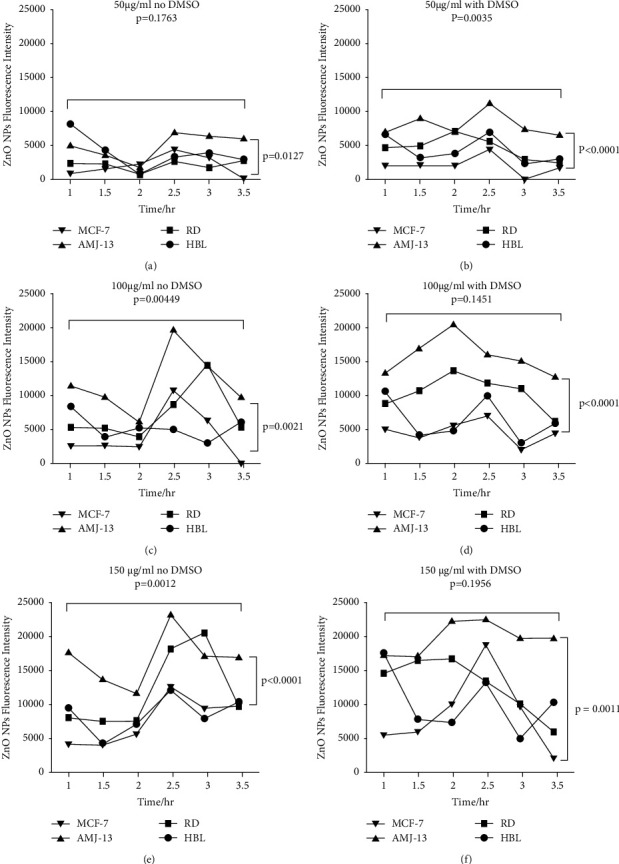
Intensity of ZnO NPs fluorescence.

The findings revealed that values of 150 and 100 ug/ml are attractive to 50 ug/ml, as shown in Figure 4, in which normal cells (HBL) had decreased sensitivity for being identified at different periods of time if contrasted to tumor cells. Additionally, when the different time periods were compared, incubation for 2.30 and 3 h contributed to the highest fluorescence intensity including all tumor cells, but incubation for 1.30 and 2 h proved to be insufficient for maximal fluorescence. The cells loaded with 150 ug/ml produced the majority of the fluorescence intensity. The fluorescence intensity was much decreased after 3.30 h compared to 2.30 h after incubation, probably owing to ZnO NP dissociation.  Figure 5 demonstrates that every concentration of ZnO NPs may be fluorescent in tumor cells preferably in normal cells. The observations revealed that DMSO had no improved impact on increasing ZnO NPs absorption into cells. When combined, 150 g/mL concentration after 2.3 h incubation yielded the greatest identification findings, which were used in subsequent investigations to corroborate the diagnosis process and image analysis. The maximal acceptable dosage of ZnO NPs surpassed 15 ug/ml, and no toxicity was found. The previous study [12] discovered that ZnO NPs at 100 ug/ml are nontoxic and biodegradable in vitro after 48 h. These findings are comparable to what we discovered in our research.

Previous studies [13] observed toxicity of ZnO NPs at a higher dose of 333.33 mg/kg/day in normal adult lab mice, indicating significantly greater than the doses employed in our present study. These findings demonstrated that directed ZnO NPs permeate and aggregate in tumor cells having great specificity, and the fluorescence intensity was stronger than that of normal cells. Comparable observations were achieved by utilizing SSEA-4 directed ZnO nanorods [14] that were discovered to attach preferentially to tumor cells and raise the photoluminescence signal significantly when compared to the control samples signal. Furthermore, they discovered that the intensity of photoluminescence increased in association with the malignant tumors for cells [14]. Nanoparticles utilized as nanoprobes for tumor-targeted visualization in vitro and in vivo were highly effective [15]. Inorganic nanoparticles have been widely employed in the detection of pancreas and breast cancer [15, 16]. The findings indicate that almost all concentrations of ZnO NPs could identify cancer cells, although normal cells (HBL) had lower fluorescence intensity to be identified at all-time intervals than tumor cells.

This finding was corroborated by previous research on the specificity of ZnO NPs in accumulating within tumor cells, which resulted in improved identification when the ZnO emitting capacity was monitored by a particular system as reported by [17, 18] which proved the high exhibition of blue emission for ZnO NPs carried out by sol-gel. Furthermore, incubation for 2.30 and 3 h yielded the highest fluorescence intensity on all tumor cells. The fluorescence signal was much decreased after 3.30 h compare to 2.30 h after incubation, most probably owing to ZnO NP dissociation. Using fluorescent data obtained from grown breast cancer cells, [19] discovered that the best intensity occurs after 3 hr. There is a study published recently [20]. In this study, researchers develop an immunosensor based on ZnO NPs for the detection of liver cancer. Another study used interdigitated electrodes nanobiosensor enhanced by AuNPs capped with ZnO NPs for detecting viral oncogenes papillomavirus subtype-16 (HPV-16) by spotting viral E6 gene targets as little as 1 FM [21]. Recently [22, 23], these studies used electrodes composed of glassy carbon, and the ZnO NPs interface was developed for electrochemical detection to diagnose breast cancer. The results propose that this biosensor could be applied in clinical practice to detect breast cancer. Mesoporous ZnO nanofibers (ZnOnF) were created for the detection of breast cancer by using an electrospinning technique with a diameter in a range of 50–150 nm of ZnO NPs [24]. K262 is leukemic cell cancer that was diagnosed after being treated for 20 minutes with ZnO-nanosheets which were synthesized by using zinc nitrate and triethanolatesumine at neutral pHand low temperature. The cells secreted a yellow-orange light, representing that ZnO nanostructures entered the cells [25]. Therefore, it is possible that NP-mediated induction of ROS may occur at or near the cell surface, and all that is required is the presence of intact particles rather than substantial NP absorption in order for this to take place. It is also likely that the sampling times affecting ZnO NPs cellular uptake trials did not cover the optimal time frame for particle uptake. Despite this, there are many mechanism (s) controlling NP cytotoxicity that is multifactorial and likely interrelated with a variety of physical parameters. These physical parameters include electrostatics, particle stability, NPs' positive or negative surface charge, agglomeration, cancer and normal cell wall integrity, and the ability to induce ROS [26].

## 4. Conclusion

Nanotechnology has the potential to remarkably improve the diagnosis of cancer cells. ZnO NPs have various characteristics that make them attractive to be used in many medical applications like a cancer diagnosis. Nanoparticles have unique characteristics such as high surface area, biologically relevant size, and enhanced permeability and retention (EPR) effect. Therefore, here we studied in vitro applications of ZnO NPs and their beneficial use as a diagnostic tool for cancer cell lines rather than normal cells. Targeting ZnO NPs can be used as good targeting NPs for the development of imaging agents for early diagnosis of cancers.

## Data Availability

The data used to support the findings of this study are included within the article.
